# Trajectories of Body Mass Index and Waist Circumference in Relation to the Risk of Cardiac Arrhythmia: A Prospective Cohort Study

**DOI:** 10.3390/nu16050704

**Published:** 2024-02-29

**Authors:** Liming Zhang, Shuohua Chen, Xingqi Cao, Jiening Yu, Zhenqing Yang, Zeinab Abdelrahman, Gan Yang, Liang Wang, Xuehong Zhang, Yimin Zhu, Shouling Wu, Zuyun Liu

**Affiliations:** 1 Second Affiliated Hospital, and School of Public Health, The Key Laboratory of Intelligent Preventive Medicine of Zhejiang Province, Zhejiang University School of Medicine, Hangzhou 310058, China; 17854238313@163.com (L.Z.); xingqi.cao@outlook.com (X.C.); jieningyu@zju.edu.cn (J.Y.); zhenqingyang@outlook.com (Z.Y.); ganyang117@outlook.com (G.Y.); 2 Department of Cardiology, Kailuan General Hospital, Hebei United University, Tangshan 063000, China; sch01062011@163.com; 3 Centre for Public Health, Queen’s University of Belfast, Belfast BT12 6BA, UK; z.abdelrahman@qub.ac.uk; 4 Department of Public Health, Robbins College of Human Health and Sciences, Baylor University, Waco, TX 76711, USA; liang_wang1@baylor.edu; 5 Department of Nutrition, Harvard T.H. Chan School of Public Health; Channing Division of Network Medicine, Brigham and Women’s Hospital and Harvard Medical School, Boston, MA 02115, USA; xuehong.zhang@channing.harvard.edu; 6 Department of Epidemiology and Biostatistics, School of Public Health, Zhejiang University, Hangzhou 310058, China; zhuym@zju.edu.cn

**Keywords:** trajectory, body mass index, waist circumference, cardiac arrhythmia, longitudinal study

## Abstract

Background: The aim of the current study was to explore the trajectories, variabilities, and cumulative exposures of body mass index (BMI) and waist circumference (WC) with cardiac arrhythmia (CA) risks. Methods: In total, 35,739 adults from the Kailuan study were included. BMI and WC were measured repeatedly during the 2006–2010 waves. CA was identified via electrocardiogram diagnosis. BMI and WC trajectories were fitted using a group-based trajectory model. The associations were estimated using Cox proportional hazards models. Results: We identified four stable trajectories for BMI and WC, respectively. Neither the BMI trajectories nor the baseline BMI values were associated with the risk of CA. Compared to the low-stable WC group, participants in the high-stable WC group had a higher risk of CA (hazard ratio (HR) = 1.40, 95% confidence interval (CI): 1.06, 1.86). Interestingly, the cumulative exposures of BMI and WC instead of their variabilities were associated with the risk of CA. In the stratified analyses, the positive associations of the high-stable WC group with the risk of CA were found in females only (HR = 1.98, 95% CI: 1.02, 3.83). Conclusions: A high-stable WC trajectory is associated with a higher risk of CA among Chinese female adults, underscoring the potential of WC rather than BMI to identify adults who are at risk.

## 1. Introduction

The electrocardiogram (ECG) abnormality of cardiac arrhythmia (CA) has gained significant attention due to its associated public health burden and its common occurrence in clinical practice. The statistical report from the American Heart Association, updated in 2021, indicates that in 2018, CA caused a total of 564,182 deaths [1]. Furthermore, atrial fibrillation (AF), the major subtype of CA, has impacted over 33 million individuals globally. It is expected that this figure will more than double in the next four decades [2,3]. The emergence of CA can trigger the occurrence of blood clots, elevating the likelihood of life-threatening complications such as severe stroke [4], myocardial infarction [5], and heart failure [6], as previously outlined. The substantial health implications stemming from the adverse effects of CA necessitate the identification of its modifiable risk factors.

The presence of obesity is a critical risk factor for the development of cardiovascular disease (CVD). The increase in adipose tissue can result in modifications in vascular hemodynamics and anomalous cardiac structural functions [7,8], thereby potentially contributing to CA development [9,10]. Prior research has investigated the correlation between obesity, predominantly measured by body mass index (BMI), and the occurrence of AF [11,12,13,14,15,16,17,18]. Waist circumference (WC) is increasingly utilized as a measure for evaluating the accumulation of abdominal visceral adipose tissue relative to BMI [19] and therefore has the potential to indicate an individual’s authentic obesity status. Simultaneously, previous studies have indicated that WC may perform better in predicting chronic diseases, including CVD [20,21]. Despite this, only a handful of studies have investigated the effect of WC on CA. Additionally, the measurement of obesity status at a single time point, as in most prior studies, fails to capture the dynamic nature of obesity status and distinguish individuals with varying patterns of obesity across time. Consequently, we hypothesized that the trajectories of different obesity indicators would have different performances in identifying the high-risk populations of CA.

Using the distinctive data of repeated measurements of BMI and WC from the Kailuan study, this longitudinal study aimed to (1) ascertain the dynamic trajectories of both BMI and WC and (2) investigate their associations with the risk of incident CA. The findings may provide a theoretical basis for the targeted health management of different types of obesity in preventing the early risk of CA and, eventually, promoting cardiovascular health.

## 2. Methods

### 2.1. Study Population

The data utilized in this study were acquired from the Kailuan study, a longitudinal cohort study established between June 2006 and October 2007. In the initial 2006 wave survey, a total of 155,418 employees and retirees from the Kailuan Group, a coal mining company in Tangshan, China, were invited to participate, of which 101,510 participants aged over 18 years provided informed consent and completed the investigation. The participants underwent biennial follow-up assessments. Comprehensive data including participant demographic characteristics, disease history, lifestyles, and biomedical indicators were collected via questionnaires, physical examinations, and laboratory tests. Further elaboration regarding the overall study design of the Kailuan study has been detailed elsewhere [22,23]. The Kailuan study was granted ethical approval by the Ethics Committee of the Kailuan Medical Group (registration number: ChiCTR2000029767) and adhered to the principles of the Declaration of Helsinki.

**Figure 1:**
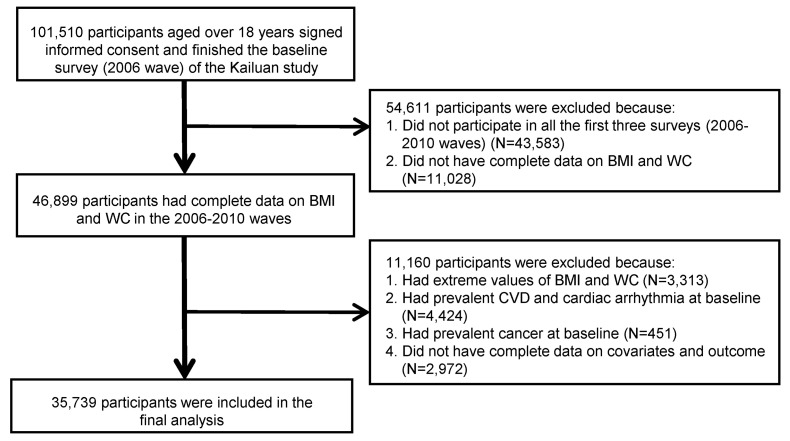
Flow chart for selecting eligible participants from the Kailuan study. Note: BMI, body mass index; WC, waist circumference; CVD, cardiovascular disease.

The trajectories were fitted by utilizing repeated data on BMI and WC from the initial three surveys (waves of 2006, 2008, and 2010). As a result, the baseline for our analysis was set as the 2010 wave. Throughout the 2006–2010 waves, BMI and WC measurements were completed by a total of 46,899 participants. We excluded participants with extreme values of BMI and WC (exceeded the 99 percentile of the overall study population, N = 3313); with prevalent CVD, CA (N = 4424), and cancer (N = 451) in and before baseline; and those who did not have complete data on covariates and outcomes (N = 2972). We ascertained CVD and cancer through the linked hospital admissions database of the Kailuan Medical Group and self-reported physician-diagnosed records [24]. The final analysis comprised 35,739 eligible participants, as shown in Figure 1.

### 2.2. Measurements of BMI and WC

During each survey, anthropometric parameters including weight, height, and WC were measured by trained field investigators. The calibrated weight and height scale was used to measure the weight (in kg) and height (in cm) of participants dressed in light clothing and without shoes. The measurement of WC (in cm) was obtained using a tape measure. All measurements were precise up to the first decimal point. The calculation of BMI (in kg/m^2^) involved dividing the weight (kg) by the square of height (m^2^).

### 2.3. Diagnosis of Cardiac Arrhythmia (CA)

Competent physicians diagnosed CA based on the ECG performance of the participants. The major subtypes of CA included atrial premature beats, atrial flutter, AF, supraventricular tachycardia, premature ventricular contraction, junctional premature beat, and non-sustained ventricular tachycardia. Then, the included participants (N = 35,739) were followed-up every biennial year up to 31 December 2020.

### 2.4. Covariates

All covariates were obtained during the baseline assessment. Standardized questionnaires were used to collect demographic information such as age (in years), sex, marital status, educational level, monthly income, drinking and smoking status, physical activity, sedentary time, and family history of CVD. The classification of marital status was divided into two categories: currently married and unmarried. Educational level was distinguished into three categories: up to primary school, middle to high school, and college or above. The categorization of the monthly income was divided into two groups: those earning ≤ 1000 Chinese yuan (CNY) and those earning > CNY 1000. Smoking and drinking statuses were defined as never, quit, and current. Physical activity frequency was classified into categories of none, occasional, and always. The duration of sedentary time was classified into three categories: less than four hours per day, between four to eight hours per day, and more than eight hours per day. In addition, various cardiometabolic indicators were evaluated, including fasting blood glucose (FBG), high-density lipoprotein (HDL), low-density lipoprotein (LDL), total cholesterol (TC), triglycerides (TG), diastolic blood pressure (DBP), and systolic blood pressure (SBP). The hexokinase/glucose-6-phosphate dehydrogenase method was utilized for FBG detection. The enzymatic colorimetric method was employed to measure HDL, LDL, TC, and TG. The measurement of DBP and SBP was carried out via the use of a mercury sphygmomanometer.

### 2.5. Statistical Analysis

**Figure 2:**
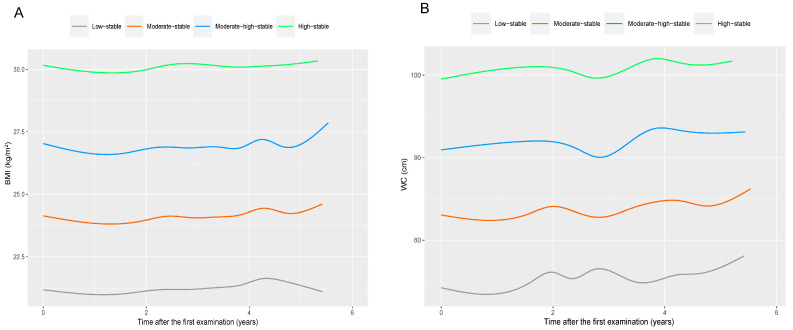
Four identified trajectories of BMI (**A**) and WC (**B**) during the 2006–2010 waves in total participants. Note: BMI, body mass index; WC, waist circumference. The mean BMI values at baseline for the low-stable BMI group, moderate-stable BMI group, moderate-high-stable BMI group, and high-stable BMI group were 21.4 kg/m^2^, 24.3 kg/m^2^, 27.0 kg/m^2^, and 30.1 kg/m^2^, respectively. The mean WC values at baseline for the low-stable WC group, moderate-stable WC group, moderate-high-stable WC group, and high-stable WC group were 75.3 cm, 84.5 cm, 93.3 cm, and 101.7 cm, respectively.

The group-based trajectory model (GBTM) was utilized to fit the trajectories of BMI and WC. This model is a finite hybrid model that identifies sub-populations with similar characteristics [25]. The selection of the best-fitted trajectory model and the number of trajectory groups was based on the Bayesian information criterion (BIC) and the average posterior probability. We identified four trajectories for BMI and WC, respectively, and designated them as the low-stable group, moderate-stable group, moderate-high-stable group, and high-stable group, as illustrated in Figure 2.

The participants’ baseline characteristics are presented as median with interquartile range (IQR) for continuous variables and numbers with percentages for categorical variables, both overall and within different trajectories. The distinctions in attributes among groups were scrutinized using the chi-square (χ^2^) test and Kruskal–Wallis tests for categorical and continuous variables, respectively.

In order to investigate the potential factors affecting BMI and WC trajectories, multinomial logistic regression models were employed to derive odds ratios (ORs) with corresponding 95% confidence intervals (CIs) for the following factors: age, sex, marital status, education level, monthly income, drinking and smoking statuses, physical activity, and sedentary time.

The primary analysis involved an examination of the relationship between the baseline BMI and WC values and the risk of CA. Participants were categorized into four quartile groups based on their baseline BMI and WC values, with the reference group being the lowest quartile group. Cox proportional hazards regression models were utilized to determine hazard ratios (HRs) with corresponding 95% CIs for the remaining three groups. Three models were fitted. Model 1 was a crude model. Model 2 was adjusted for age and sex. Model 3 was further adjusted for marital status, educational level, monthly income, smoking status, drinking status, physical activity, and sedentary time based on model 2. Subsequently, Cox proportional hazards regression models were utilized to investigate the associations between the trajectories of BMI and WC and the likelihood of CA, with the low-stable group serving as the reference group.

In further analyses, we employed Cox proportional hazards regression models utilizing the restricted cubic spline to evaluate the dose–response associations of BMI and WC variabilities, which were determined by the coefficient of variation (CV) of BMI and WC in the 2006–2010 waves, as well as their cumulative exposures with the risk of CA. The exposure to cumulative BMI (cBMI) and WC (cWC) was computed by adding up the excessive BMI- and WC-years with reference to their standards (24 kg/m^2^ for BMI; 90 cm and 85 cm for WC in males and females, respectively) throughout the follow-up period.

Stratified analyses were conducted by fitting BMI and WC trajectories by sex and age sub-groups (<45 years, 45–59 years, and ≥60 years). Subsequently, the sex- or age-specific trajectories were utilized to investigate the associations between BMI and WC trajectories with CA risk in each sub-group.

In sensitivity analyses, we repeated the primary analyses for BMI and WC trajectories by (1) further adjusting for several cardiometabolic indicators, including LDL, HDL, TC, TG, SBP, and DBP (Model 4), or the family history of CVD (model 5) as based on model 3 and (2) assessing the competing risk from all-cause death (model 6) using the Fine and Gray competing risk model [26]. Statistical significance was determined by a two-sided *p*-value of less than 0.05. SAS software (version 9.4, SAS Institute, Cary, NC, USA) and R software (version 4.0.4) were employed to conduct all statistical analyses.

## 3. Results

In accordance with Figure 2, it is evident that the mean BMI values at baseline for the low-stable BMI group, moderate-stable BMI group, moderate-high-stable BMI group, and high-stable BMI group were 21.4 kg/m^2^, 24.3 kg/m^2^, 27.0 kg/m^2^, and 30.1 kg/m^2^, respectively, with each group comprising 20.7%, 38.5%, 30.6%, and 10.2% of the total participants. The low-stable WC group (comprising 12.6% of total participants), moderate-stable WC group (comprising 41.3% of total participants), moderate-high-stable WC group (comprising 37.3% of total participants), and high-stable WC group (comprising 8.8% of total participants) exhibited respective mean WC values of 75.3 cm, 84.5 cm, 93.3 cm, and 101.7 cm at baseline. Figure S1 illustrates the normal distributions of BMI and WC during the 2006–2010 waves. The baseline characteristics of study participants in total and by different trajectories are illustrated in Table 1. In brief, the median age of all participants was 52.2 years (with an interquartile range of 45.0 to 59.1), and the majority were male (77.3%). Participants belonging to the other three BMI trajectories had a greater propensity to be older and male in contrast to those in the low-stable BMI group. The WC trajectories also yielded similar outcomes.

The factors linked to BMI and WC trajectories are depicted in Table S1. Compared to participants in the low-stable BMI group, those in the other BMI trajectories were more likely to be older (moderate-stable: OR = 1.01, 95% CI: 1.01, 1.02; moderate-high-stable: OR = 1.01, 95% CI: 1.01, 1.02; high-stable: OR = 1.00, 95% CI: 1.00, 1.01); to be male (moderate-stable: OR = 1.61, 95% CI: 1.49, 1.73; moderate-high-stable: OR = 2.00, 95% CI: 1.84, 2.16; high-stable: OR = 1.50, 95% CI: 1.35, 1.67); and to have drinking habits (moderate-stable: OR = 1.11, 95% CI: 1.03, 1.20; moderate-high-stable: OR = 1.12, 95% CI: 1.04, 1.21; high-stable: OR = 1.11, 95% CI: 1.00, 1.23). Similar results were also found in different WC trajectories. Moreover, participants in the moderate-high-stable WC group and the high-stable WC group had a higher monthly income than those in the low-stable WC group (moderate-high-stable: OR = 1.25, 95% CI: 1.16, 1.35; high-stable: OR = 1.56, 95% CI: 1.41, 1.72).

The associations between baseline BMI and WC values and CA risks are displayed in Table 2. In brief, there was no significant association between BMI and the risk of CA. In contrast, compared with participants in the lowest WC quartile group, those in the highest quartile group (Q4) had a higher risk of CA (HR = 1.24, 95% CI: 1.03, 1.49 in model 3). Associations of BMI and WC trajectories with the risk of CA are shown in Table 3. No significant associations of the BMI trajectories with the risk of CA were observed. In contrast, participants in the moderate-stable, moderate-high-stable, and high-stable WC groups had a higher risk of CA in the crude model than participants in the low-stable WC group. However, after adjusting for covariates, only the high-stable WC group was associated with a higher risk of CA (HR = 1.40, 95% CI: 1.06, 1.86 in model 3).

In additional analyses, none of the variabilities of BMI and WC were significantly associated with the risk of CA (Figure S2), while both the cBMI and cWC were positively associated with high risks of CA (Figure S3).

As shown in Figures S4 and S5, the trajectories for BMI and WC in different age and sex sub-groups were similar to that in total participants. Consistent with the results in total participants, BMI trajectories were still not significantly associated with the risk of CA in age- or sex-stratified analyses. However, for WC trajectories, females in the high-stable WC group (mean WC = 100.7 cm at baseline) were at a higher risk of CA (HR = 1.98, 95% CI: 1.02, 3.83) in comparison to the non-significant associations of WC trajectories with CA in males (i.e., HR = 1.31, 95% CI: 0.98, 1.75 for the high-stable WC group) (Table S2).

In the sensitivity analyses, the results remained robust (Table S3) after adjusting for several cardiometabolic indicators, the family history of CVD, or considering the competing risk from all-cause death, respectively.

## 4. Discussion

Our research conducted on a significant group of Chinese adults reveals that the obesity status remains consistent over a short period, and variability of obesity may not be an appropriate approach to identify individuals at a high risk of CA. Subsequently, we determined four stable trajectories for both BMI and WC, and participants with the trajectory of high stability in WC and higher initial WC values were found to have an increased risk of CA. In comparison, there was no significant association between either the BMI trajectories or the initial BMI values and the risk of CA. The findings suggest that WC might be a more suitable measure to identify individuals at risk, and the monitoring and management of central obesity is likely to be an effective method of preventing the early risk of CA.

Prior research has suggested that elevated or high-stable WC trajectories are positively linked to the risk of AF and CVD [24,27]. In contrast to the previous longitudinal studies that evaluated obesity over a significantly longer period (e.g., Lu et al.’s 16-year study [27]), our results demonstrate the stability of obesity status over a shorter period (i.e., 6 years) and the appropriateness of WC as a stable marker for identifying individuals at increased risk of CA. In addition, only females displayed positive associations between high-stable WC trajectory and CA risk. It is possible that females have a greater tendency to possess higher body fat percentages even when their WC falls within the same range as that of males [28]. Additionally, studies have demonstrated that the risk of CVD is elevated in females with increasing age. The potential reason for this phenomenon could be attributed to the reduction of estrogen’s protective effects on females [29,30]. This explanation may be attributed to the fact that our study included a significant number of middle-aged females who were undergoing menopause. The results emphasize the significance of daily monitoring and management of WC among middle-aged females.

One longitudinal study supported our findings of a non-significant association between trajectories and initial values of BMI with CA, of which the researchers concluded that BMI alone may not be a reliable indicator for identifying high-risk CVD individuals among middle-aged and elderly populations [31]. Despite this, other studies have produced divergent findings. Lu et al. detected a persistent-increasing BMI trajectory and concluded that this trend was linked to a higher probability of AF [27]. Also, it was previously indicated through multiple research studies that the BMI trajectories or higher BMI values during early life stages may have an impact on later CVD risks [32,33,34,35]. The heterogeneity of results may encompass the subsequent aspects: First, the participants studied by Lu et al. were mainly older adults (mean ages for males and females were 72.3 and 73.7 years, respectively), who showed a higher risk of CVD compared to the middle-aged participants in our study. Second, a prolonged follow-up period of over 16 years was employed in their study to establish BMI trajectories, which greatly impacted their results. Our additional analyses revealed a positive correlation between cumulative BMI exposures and the high risk of CA, which emphasizes the need to monitor long-term trajectories and cumulative exposures of general obesity.

The difference in BMI and WC performance in identifying individuals at risk of CA may partially have a clinical explanation. BMI is typically used to indicate general obesity [36]. However, to assess central obesity and the accumulation of abdominal visceral adipose tissue, WC is considered a better indicator [19]. WC could potentially provide a more accurate representation of an individual’s obesity status, whereas BMI has been frequently utilized in examining the impact of obesity on CVD in previous research. Given the current evidence, we hypothesize that long-term BMI trajectories rather than short-term trajectories may aid in identifying middle-aged and elderly individuals who are at a heightened risk of CA. In the meantime, it is recommended to measure WC as opposed to relying solely on BMI, as some individuals with “normal weight” may have abdominal obesity, which can conceal their high risks of CVD [10]. In order to fully demonstrate the actual correlations and fundamental mechanisms of obesity with health hazards, it is imperative to take into account various obesity indicators from multiple perspectives, as we have done in our present investigation [37,38,39].

The potential mechanisms through which obesity affects CA may encompass the following aspects [9]: First, obesity can exert influence on the stability of hemodynamics by increasing blood volume, stroke volume, and arterial pressure, which may in turn result in CVD. The second point to consider is that obesity has the potential to cause pathophysiologic changes, including inflammatory reactions and the overexpression of tumor necrosis factor (TNF), which are significant risk factors for the development of CVD [40,41]. Third, the neurohumoral (i.e., insulin resistance and hyperinsulinemia) and cellular pathways (i.e., hypertrophy, apoptosis, and fibrosis) may also play a key role in this process.

Our study presents several strengths. Initially, this is the first study to systematically investigate the connections between BMI and WC trajectories and the probability of developing CA, and the current findings highlighted the potential of central obesity over general obesity in identifying at-risk individuals. Simultaneously, our study may also provide a theoretical basis for the targeted health management of different types of obesity in preventing early risk of CA. Second, the substantial sample size ensured the dependability of the findings. Third, the prospective nature of the design served to minimize the possibility of recall bias while ensuring that the cohort retained comprehensive information for our analysis. Fourth, we minimized the reverse causality bias by excluding participants with existing CVD and outcome events at baseline.

Despite this, we acknowledge some limitations. First, it is important to note that misclassification bias resulting from diagnostic errors may persist despite the evaluation of diagnoses by experienced medical professionals. Second, the analysis may have overlooked some other confounding factors. Third, the Kailuan study lacked national representativeness, and therefore, the conclusions may not be suitable to be extrapolated to other ethnic/genetically distinct populations. Additionally, we avoided conducting the secondary analysis for various subtypes of CA owing to the low prevalence of each subtype. In addition, certain obesity indicators that reflect body composition, such as body fat ratio, were not assessed in this cohort.

## 5. Conclusions

Among Chinese adults, the status of obesity remains constant over a relatively short period, and the use of obesity variability may not be a suitable measure for identifying individuals at high risk of CA. The study indicates that in females, higher WC values and a high-stable WC trajectory carry a greater risk of CA than BMI. Thus, WC may prove to be an effective measure for identifying at-risk females. The effective prevention of CA is expected to be achieved through the monitoring and management of central obesity.

## Figures and Tables

**Table 1:** Baseline characteristics of participants in total and by different trajectories.

		BMI Trajectory					WC Trajectory				
	Total (N = 35,739)	Low-Stable (N = 7400)	Moderate-Stable (N = 13,750)	Moderate-High- Stable (N = 10,950)	High-Stable (N = 3639)	*p*-Value	Low-Stable (N = 4489)	Moderate-Stable (N = 14,758)	Moderate-High- Stable (N = 13,352)	High-Stable (N = 3140)	*p*-Value
**Age, Years**	52.2 (45.0, 59.1)	50.3 (42.6, 57.7)	52.6 (45.7, 59.3)	52.8 (45.5, 59.7)	52.2 (44.1, 59.5)	<0.001	46.7 (39.2, 54.0)	51.9 (45.0, 58.8)	53.4 (46.4, 60.1)	54.7 (46.9, 62.5)	<0.001
**Sex**						<0.001					<0.001
Female	8123 (22.7)	2204 (29.8)	2987 (21.7)	2045 (18.7)	887 (24.4)		2309 (51.4)	3655 (24.8)	1861 (13.9)	298 (9.5)	
Male	27,616 (77.3)	5196 (70.2)	10,763 (78.3)	8905 (81.3)	2752 (75.6)		2180 (48.6)	11,103 (75.2)	11,491 (86.1)	2842 (90.5)	
**Marital status**						<0.001					<0.001
Unmarried	422 (1.2)	135 (1.8)	140 (1.0)	108 (1.0)	39 (1.1)		107 (2.4)	155 (1.1)	127 (1.0)	33 (1.1)	
Currently married	35,317 (98.8)	7265 (98.2)	13,610 (99.0)	10,842 (99.0)	3600 (98.9)		4382 (97.6)	14,603 (98.9)	13,225 (99.0)	3107 (98.9)	
**Educational level**						<0.001					<0.001
Primary school	2365 (6.6)	426 (5.8)	950 (6.9)	747 (6.8)	242 (6.7)		199 (4.4)	921 (6.2)	990 (7.4)	255 (8.1)	
Middle-high school	30,376 (85.0)	6178 (83.5)	11,727 (85.3)	9378 (85.6)	3093 (85.0)		3596 (80.1)	12,698 (86.0)	11,389 (85.3)	2693 (85.8)	
College or above	2998 (8.4)	796 (10.8)	1073 (7.8)	825 (7.5)	304 (8.4)		694 (15.5)	1139 (7.7)	973 (7.3)	192 (6.1)	
**Monthly income**						0.896					<0.001
≤CNY 1000	18,015 (50.4)	3701 (50.0)	6950 (50.5)	5530 (50.5)	1834 (50.4)		2360 (52.6)	7933 (53.8)	6398 (47.9)	1324 (42.2)	
>CNY 1000	17,724 (49.6)	3699 (50.0)	6800 (49.5)	5420 (49.5)	1805 (49.6)		2129 (47.4)	6825 (46.2)	6954 (52.1)	1816 (57.8)	
**Drinking status**						<0.001					<0.001
Never	22,835 (63.9)	4896 (66.2)	8745 (63.6)	6823 (62.3)	2371 (65.2)		3400 (75.7)	9640 (65.3)	7955 (59.6)	1840 (58.6)	
Quit	140 (0.4)	22 (0.3)	60 (0.4)	45 (0.4)	13 (0.4)		12 (0.3)	51 (0.3)	60 (0.4)	17 (0.5)	
Current	12,764 (35.7)	2482 (33.5)	4945 (36.0)	4082 (37.3)	1255 (34.5)		1077 (24.0)	5067 (34.3)	5337 (40.0)	1283 (40.9)	
**Smoking status**						<0.001					<0.001
Never	21,715 (60.8)	4508 (60.9)	8320 (60.5)	6559 (59.9)	2328 (64.0)		3307 (73.7)	9121 (61.8)	7607 (57.0)	1680 (53.5)	
Quit	1445 (4.0)	245 (3.3)	558 (4.1)	489 (4.5)	153 (4.2)		76 (1.7)	545 (3.7)	637 (4.8)	187 (6.0)	
Current	12,579 (35.2)	2647 (35.8)	4872 (35.4)	3902 (35.6)	1158 (31.8)		1106 (24.6)	5092 (34.5)	5108 (38.3)	1273 (40.5)	
**Physical activity**						<0.001					<0.001
None	11,262 (31.5)	2500 (33.8)	4292 (31.2)	3367 (30.7)	1103 (30.3)		1567 (34.9)	4579 (31.0)	4102 (30.7)	1014 (32.3)	
Occasional	19,731 (55.2)	3987 (53.9)	7658 (55.7)	6028 (55.1)	2058 (56.6)		2427 (54.1)	8244 (55.9)	7388 (55.3)	1672 (53.2)	
Always	4746 (13.3)	913 (12.3)	1800 (13.1)	1555 (14.2)	478 (13.1)		495 (11.0)	1935 (13.1)	1862 (13.9)	454 (14.5)	
**Sedentary time per day**						<0.001					<0.001
<4 h	16,761 (46.9)	3461 (46.8)	6612 (48.1)	5036 (46.0)	1652 (45.4)		1852 (41.3)	6903 (46.8)	6411 (48.0)	1595 (50.8)	
4–8 h	17,804 (49.8)	3677 (49.7)	6741 (49.0)	5522 (50.4)	1864 (51.2)		2444 (54.4)	7387 (50.1)	6523 (48.9)	1450 (46.2)	
>8 h	1174 (3.3)	262 (3.5)	397 (2.9)	392 (3.6)	123 (3.4)		193 (4.3)	468 (3.2)	418 (3.1)	95 (3.0)	

Note: BMI, body mass index; WC, waist circumference; CNY, Chinese yuan. The baseline characteristics of participants are expressed as the median (interquartile range) for continuous variables and the participant number (percentages) for categorical variables, respectively. The *p*-values were calculated using χ^2^ and Kruskal–Wallis tests for categorical and continuous variables, respectively.

**Table 2:** Associations of the BMI and WC values at baseline with the risk of cardiac arrhythmia.

**BMI Quartile Groups**				
	**Q1** **(N = 8956)**	**Q2** **(N = 8792)**	**Q3** **(N = 9023)**	**Q4** **(N = 8968)**
BMI values at baseline, kg/m^2^	21.4 (17.6, 22.9)	24.0 (22.9, 24.9)	26.0 (24.9, 27.1)	29.0 (27.1, 35.6)
Cases/Person-years	233/88,963	251/87,064	258/89,413	278/88,634
Model 1	Ref.	1.10 (0.92, 1.32)	1.10 (0.92, 1.32)	1.20 (1.01, 1.43) *
Model 2	Ref.	1.05 (0.88, 1.25)	1.04 (0.87, 1.24)	1.16 (0.98, 1.38)
Model 3	Ref.	1.04 (0.87, 1.25)	1.04 (0.87, 1.24)	1.15 (0.97, 1.37)
**WC Quartile Groups**				
	**Q1** **(N = 8488)**	**Q2** **(N = 8403)**	**Q3** **(N = 8836)**	**Q4** **(N = 10,012)**
WC values at baseline, cm	76.2 (62.0, 81.7)	84.7 (82.0, 87.2)	90.4 (88.0, 93.7)	99.2 (94.0, 114.0)
Cases/Person-years	85/84,373	409/83,291	397/87,614	129/98,795
Model 1	Ref.	1.35 (1.11, 1.64) *	1.40 (1.16, 1.69) *	1.63 (1.36, 1.96) *
Model 2	Ref.	1.14 (0.94, 1.39)	1.12 (0.92, 1.36)	1.22 (1.01, 1.46) *
Model 3	Ref.	1.15 (0.95, 1.40)	1.13 (0.93, 1.37)	1.24 (1.03, 1.49) *

Note: BMI, body mass index; WC, waist circumference; Q, quartile. BMI and WC values at baseline were expressed as the median (interquartile range). The Cox proportional hazards regression models were used to examine the associations of BMI and WC values with the risk of cardiac arrhythmia, and the hazard ratios with the corresponding 95% confidence intervals were calculated. Model 1 was a crude model; model 2 was adjusted for age and sex; model 3 was further adjusted for marital status, educational level, monthly income, smoking status, drinking status, physical activity, and sedentary time based on model 2. * indicates statistical significance (*p* < 0.05).

**Table 3:** Associations of BMI and WC trajectories with the risk of cardiac arrhythmia.

**BMI Trajectory**				
	**Low-Stable** **(N = 7400)**	**Moderate-Stable** **(N = 13,750)**	**Moderate-High-Stable** **(N = 10,950)**	**High-Stable** **(N = 3639)**
Mean BMI values at baseline, kg/m^2^	21.4	24.3	27.0	30.1
Cases/Person-years	194/73,405	376/136,257	339/108,368	111/36,043
Model 1	Ref.	1.05 (0.88, 1.24)	1.18 (0.99, 1.41)	1.17 (0.92, 1.47)
Model 2	Ref.	0.96 (0.81, 1.15)	1.09 (0.91, 1.30)	1.14 (0.90, 1.44)
Model 3	Ref.	0.96 (0.80, 1.14)	1.08 (0.90, 1.29)	1.12 (0.89, 1.42)
**WC Trajectory**				
	**Low-Stable** **(N = 4489)**	**Moderate-Stable** **(N = 14,758)**	**Moderate-High-Stable** **(N = 13,352)**	**High-Stable** **(N = 3140)**
Mean WC values at baseline, cm	75.3	84.5	93.3	101.7
Cases/Person-years	85/44,653	409/146,225	397/132,325	129/30,870
Model 1	Ref.	1.47 (1.16, 1.85) *	1.58 (1.25, 1.99) *	2.20 (1.67, 2.89) *
Model 2	Ref.	1.10 (0.87, 1.40)	1.08 (0.85, 1.37)	1.36 (1.03, 1.82) *
Model 3	Ref.	1.11 (0.88, 1.41)	1.09 (0.86, 1.39)	1.40 (1.06, 1.86) *

Note: BMI, body mass index; WC, waist circumference. The Cox proportional hazards regression models were used to examine the associations of BMI and WC trajectories with the risk of cardiac arrhythmia, and the hazard ratios with the corresponding 95% confidence intervals were calculated. Model 1 was a crude model; model 2 was adjusted for age and sex; model 3 was adjusted for marital status, educational level, monthly income, smoking status, drinking status, physical activity, and sedentary time based on model 2. * indicates statistical significance (*p* < 0.05).

## Data Availability

The data presented in this study are available on request from the corresponding author. The data are not publicly available due to privacy.

## Supplementary Materials

The following supporting information can be downloaded at: https://www.mdpi.com/article/10.3390/nu16050704/s1, Figure S1: Distributions of BMI (A) and WC (B) in the 2006-2010 waves; Figure S2: Dose-response relationships of BMI (A) and WC (B) variabilities with the risk of cardiac arrhythmia; Figure S3: Dose-response relationships of the cumulative BMI (A) and WC (B) exposures with the risk of cardiac arrhythmia; Figure S4: Four trajectories of BMI (A) and WC (B) were identified in age-specific subgroups; Figure S5: Four trajectories of BMI (A) and WC (B) were identified in sex-specific subgroups; Table S1: Factors associated with BMI and WC trajectories, respectively; Table S2: Associations of BMI and WC trajectories with the risk of cardiac arrhythmia in stratified analyses by age and sex; Table S3: Sensitivity analyses for the associations of BMI and WC trajectories with the risk of cardiac arrhythmia.

## Abbreviations

BMI, body mass index; WC, waist circumference; CA, cardiac arrhythmia; AF, atrial fibrillation; ECG, electrocardiogram; CVD, cardiovascular disease; HR, hazard ratio; OR, odds ratio; CI, confidence interval.
