# Supplementary material for: Trajectories of Body Mass Index and Waist Circumference in Relation to the Risk of Cardiac Arrhythmia: A Prospective Cohort Study
Source: Nutrients. 2024 Feb 29;16(5):704. doi: 10.3390/nu16050704 (PMC10934224; doi:10.3390/nu16050704)
Supplement: Supplementary file 1 [file nutrients-16-00704-s001.zip › nutrients-2858412-supplementary.pdf]

Trajectories of Body Mass Index and Waist Circumference in Relation to the Risk of Cardiac Arrhythmia: A Prospective Cohort Study

Liming Zhang [1,†], Shuohua Chen [2,†], Xingqi Cao [1,†], Jiening Yu [1,†], Zhenqing Yang [1], Zeinab Abdelrahman [3], Gan Yang [1], Liang Wang [4], Xuehong Zhang [5], Yimin Zhu [6], Shouling Wu [2,*] and Zuyun Liu [1,*]

[1] Second Affiliated Hospital, and School of Public Health, The Key Laboratory of Intelligent Preventive Medicine of Zhejiang Province, Zhejiang University School of Medicine, Hangzhou 310058, China; 17854238313@163.com (L.Z.); xingqi.cao@outlook.com (X.C.); jieningyu@zju.edu.cn (J.Y.); zhenqingyang@outlook.com (Z.Y.); ganyang117@outlook.com (G.Y.)
[2] Department of Cardiology, Kailuan General Hospital, Hebei United University, Tangshan 063000, China; sch01062011@163.com
[3] Centre for Public Health, Queen's University of Belfast, Belfast BT12 6BA, UK; z.abdelrahman@qub.ac.uk
[4] Department of Public Health, Robbins College of Human Health and Sciences, Baylor University, Waco, TX 76711, USA; liang_wang1@baylor.edu
[5] Department of Nutrition, Harvard T.H. Chan School of Public Health; Channing Division of Network Medicine, Brigham and Women's Hospital and Harvard Medical School, Boston, MA 02115, USA; xuehong.zhang@channing.harvard.edu
[6] Department of Epidemiology and Biostatistics, School of Public Health, Zhejiang University, Hangzhou 310058, China; zhuym@zju.edu.cn
* Correspondence: zuyun.liu@outlook.com or zuyunliu@zju.edu.cn (Z.L.); drwusl@163.com (S.W.); Tel.: +86-0571-8707-7127 (Z.L.)
† These authors contributed equally to this work.

Figure S1. Distributions of BMI (A) and WC (B) in the 2006-2010 waves.

Figure S2. Dose-response relationships of BMI (A) and WC (B) variabilities with the risk of cardiac arrhythmia.

Figure S3. Dose-response relationships of the cumulative BMI (A) and WC (B) exposures with the risk of cardiac arrhythmia.

Figure S4. Four trajectories of BMI (A) and WC (B) were identified in age-specific subgroups.

Figure S5. Four trajectories of BMI (A) and WC (B) were identified in sex-specific subgroups.

Table S1. Factors associated with BMI and WC trajectories, respectively.

Table S2. Associations of BMI and WC trajectories with the risk of cardiac arrhythmia in stratified analyses by age and sex.

Table S3. Sensitivity analyses for the associations of BMI and WC trajectories with the risk of cardiac arrhythmia.

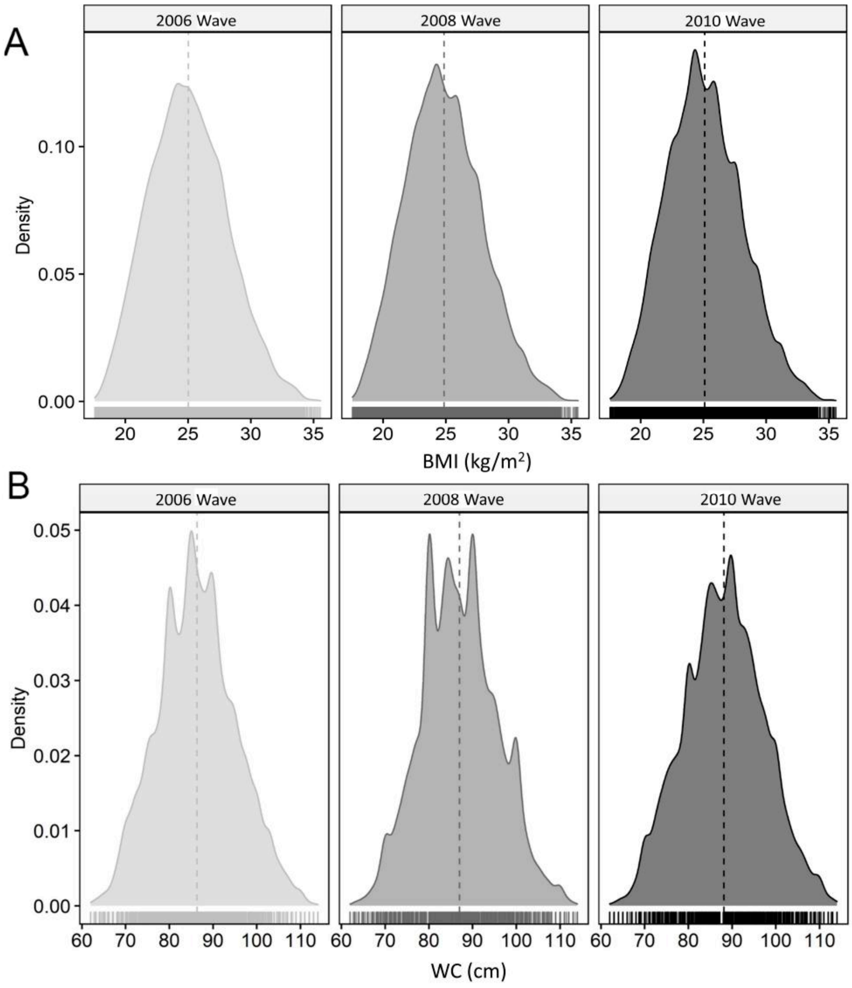

Figure S1. Distributions of BMI (A) and WC (B) in the 2006-2010 waves.

Note: BMI, body mass index; WC, waist circumference.

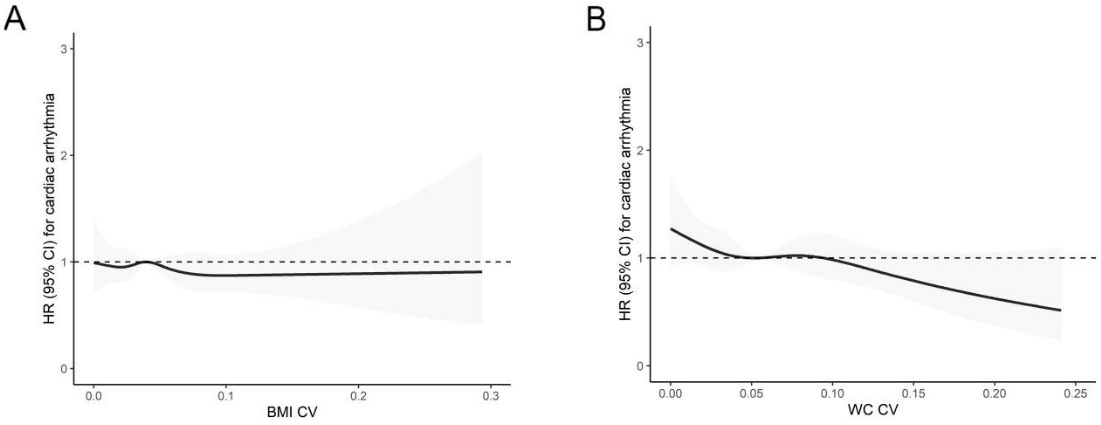

Figure S2. Dose-response relationships of BMI (A) and WC (B) variabilities with the risk of cardiac arrhythmia.

Note: BMI, body mass index; WC, waist circumference; HR, Hazard Ratio; CI, confidence interval; CV, coefficient of variation. The Cox proportional hazards regression models with the Restricted Cubic Spline were used to examine the dose-response associations of BMI and WC variabilities with the risk of cardiac arrhythmia, adjusted for age, sex, marital status, educational level, monthly income, smoking status, drinking status, physical activity, and sedentary time.

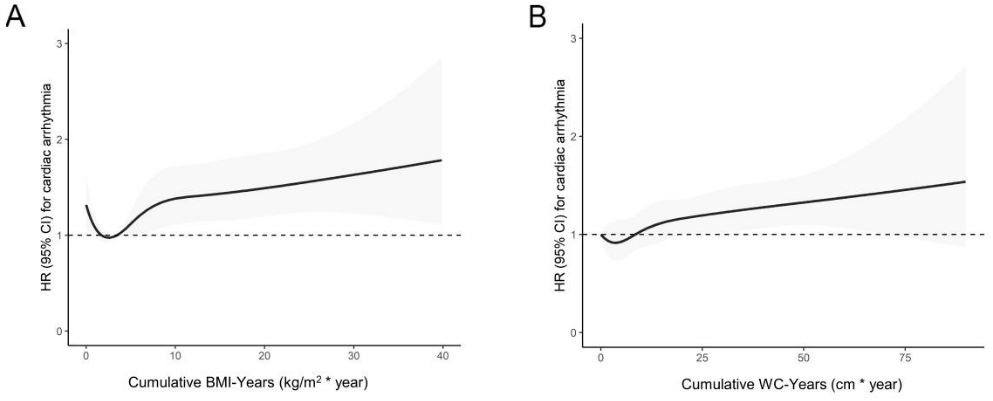

Figure S3. Dose-response relationships of the cumulative BMI (A) and WC (B) exposures with the risk of cardiac arrhythmia.

Note: BMI, body mass index; WC, waist circumference; HR, Hazard Ratio; CI, confidence interval。

The Cox proportional hazards regression models with the Restricted Cubic Spline were used to examine the dose-response associations of the cumulative BM and WC exposures with the risk of cardiac arrhythmia, adjusted for age, sex, marital status, educational level, monthly income, smoking status, drinking status, physical activity, and sedentary time.

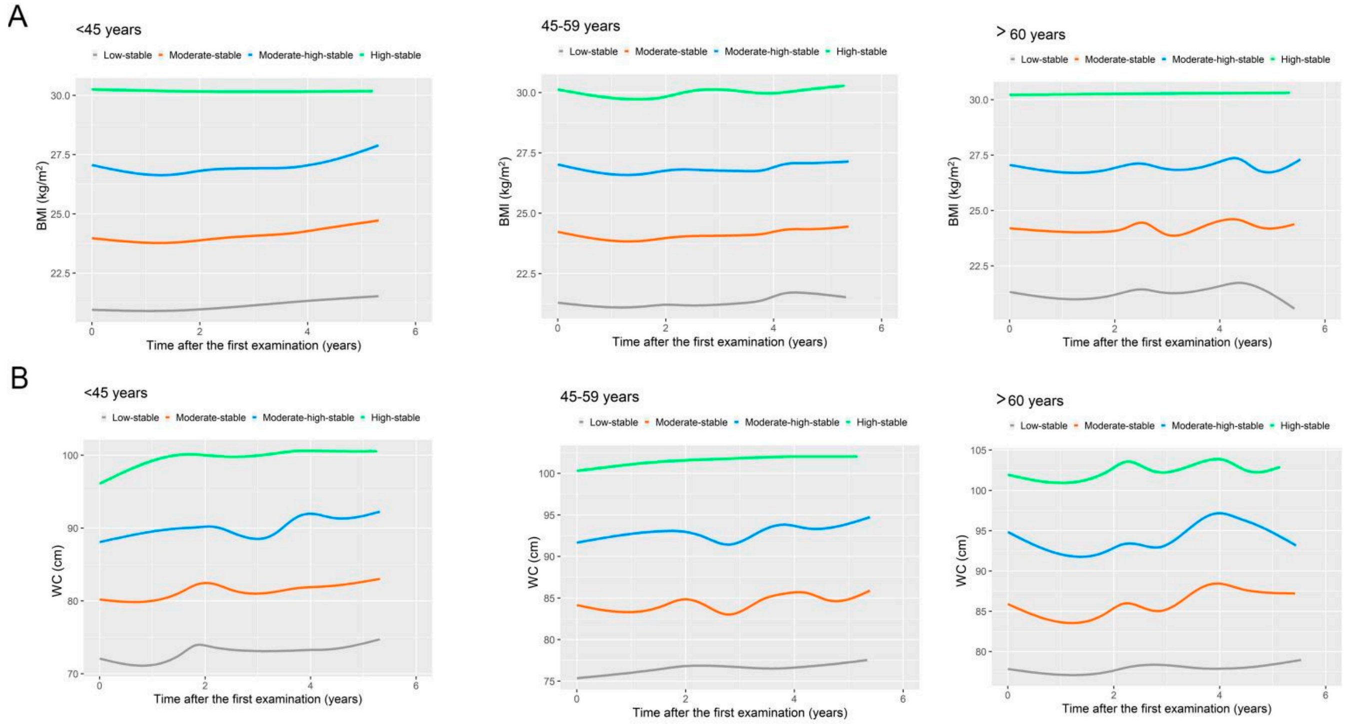

Figure S4. Four trajectories of BMI (A) and WC (B) were identified in age-specific subgroups.

Note: BMI, body mass index; WC, waist circumference.

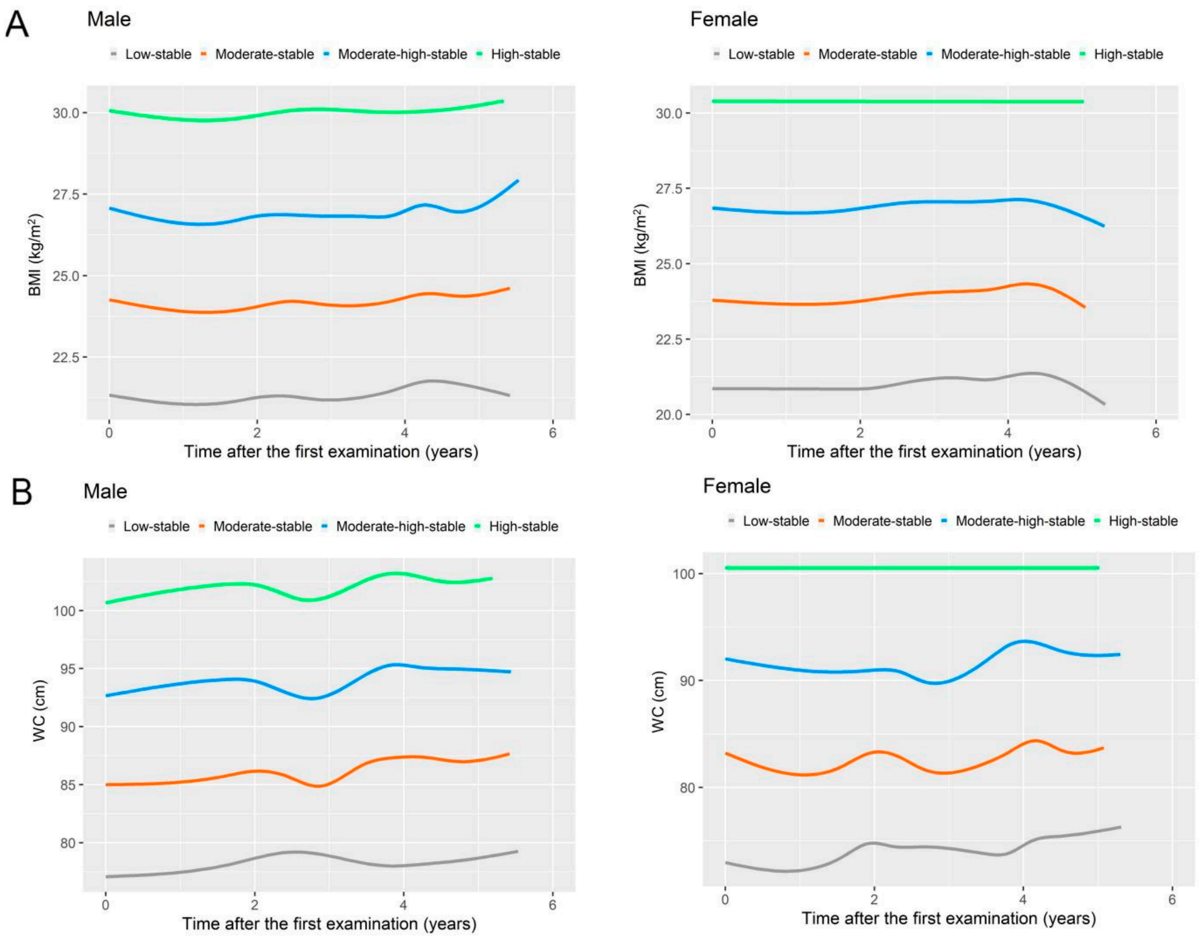

Figure S5. Four trajectories of BMI (A) and WC (B) were identified in sex-specific subgroups.

Note: BMI, body mass index; WC, waist circumference.

Table S1. Factors associated with BMI and WC trajectories, respectively.

	BMI trajectory				WC trajectory			
	Low-stable (N=7,400)	Moderate-stable (N=13,750)	Moderate-high-Stable (N=10,950)	High-stable (N=3,639)	Low-stable (N=4,489)	Moderate-stable (N=14,758)	Moderate-high-Stable (N=13,352)	High-stable (N=3,140)
**Age, Years**	Ref.	1.01 (1.01, 1.02)*	1.01 (1.01, 1.02)*	1.00 (1.00, 1.01)*	Ref.	1.04 (1.04, 1.05)*	1.05 (1.05, 1.06)*	1.07 (1.06, 1.07)*
**Sex**	Ref.	1.61 (1.49, 1.73)*	2.00 (1.84, 2.16)*	1.50 (1.35, 1.67)*	Ref.	3.32 (3.04, 3.63)*	6.76 (6.15, 7.43)*	10.28 (8.86, 11.94)*
**Marital status**	Ref.	1.61 (1.27, 2.05)*	1.67 (1.29, 2.16)*	1.64 (1.15, 2.36)*	Ref.	1.72 (1.32, 2.23)*	1.78 (1.34, 2.36)*	1.54 (1.01, 2.33)*
**Monthly income**	Ref.	0.98 (0.92, 1.04)	0.98 (0.92, 1.04)	1.01 (0.93, 1.10)	Ref.	0.98 (0.91, 1.05)	1.25 (1.16, 1.35)*	1.56 (1.41, 1.72)*
**Educational level**								
Middle -high school	Ref.	0.94 (0.83, 1.06)	0.97 (0.86, 1.11)	0.90 (0.76, 1.06)	Ref.	1.07 (0.91, 1.26)	1.02 (0.86, 1.20)	1.05 (0.86, 1.29)
College or above	Ref.	0.77 (0.66, 0.90)	0.75 (0.64, 0.89)	0.69 (0.55, 0.85)	Ref.	0.79 (0.65, 0.97)	0.76 (0.62, 0.94)	0.71 (0.54, 0.93)
**Drinking status**								
Quit	Ref.	1.36 (0.83, 2.23)	1.20 (0.72, 2.02)	1.16 (0.58, 2.33)	Ref.	0.75 (0.39, 1.43)	0.84 (0.44, 1.59)	0.84 (0.39, 1.80)
Current	Ref.	1.11 (1.03, 1.20)*	1.12 (1.04, 1.21)*	1.11 (1.00, 1.23)*	Ref.	1.14 (1.03, 1.26)*	1.28 (1.16, 1.42)*	1.23 (1.09, 1.40)*
**Smoking status**								
Quit	Ref.	0.97 (0.82, 1.14)	0.98 (0.83, 1.16)	0.96 (0.77, 1.19)	Ref.	1.40 (1.09, 1.81)*	1.53 (1.19, 1.97)*	1.95 (1.46, 2.60)*
Current	Ref.	0.82 (0.76, 0.88)	0.76 (0.70, 0.82)	0.68 (0.61, 0.76)	Ref.	0.96 (0.87, 1.07)	0.93 (0.84, 1.03)	1.05 (0.92, 1.19)
**Physical activity**								
Occasional	Ref.	1.11 (1.04, 1.19)*	1.14 (1.06, 1.22)*	1.19 (1.09, 1.31)*	Ref.	1.19 (1.10, 1.29)*	1.25 (1.15, 1.35)*	1.14 (1.02, 1.28)*
Always	Ref.	1.08 (0.98, 1.19)	1.19 (1.08, 1.32)*	1.17 (1.02, 1.34)*	Ref.	1.18 (1.05, 1.33)*	1.21 (1.07, 1.36)*	1.12 (0.96, 1.31)
**Sedentary time, Per day**								
4-8 hours	Ref.	1.03 (0.97, 1.09)	1.12 (1.05, 1.19)*	1.15 (1.06, 1.25)*	Ref.	0.91 (0.85, 0.98)	0.85 (0.79, 0.92)	0.73 (0.66, 0.81)

> 8 hours	Ref.	0.95 (0.81, 1.13)	1.27 (1.08, 1.50)*	1.15 (0.92, 1.44)	Ref.	0.99 (0.82, 1.20)	1.02 (0.84, 1.24)	0.97 (0.74, 1.27)

Note: BMI, body mass index; WC, waist circumference.

The multinomial logistic regression models were used to explore the factors associated with BMI and WC trajectories, and odds ratios with the corresponding 95% confidence intervals were calculated, including age (in years), sex (female as reference), marital status (unmarried as reference), monthly income (<1000 CNY as reference), educational level (up to primary school as reference), drinking status (never drinking as reference), smoking status (never smoking as reference), physical activity (none as reference), and sedentary time (< 4 hours per day as reference)

* indicated statistical significance (P<0.05).

Table S2. Associations of BMI and WC trajectories with the risk of cardiac arrhythmia in stratified analyses by age and sex.

	BMI trajectory				WC trajectory			
	**Low-stable**	**Moderate-stable**	**Moderate-high stable**	**High-stable**	**Low-stable**	**Moderate-stable**	**Moderate-high-stable**	**High-stable**
**Age**								
**<45 years**								
Mean BMI/WC values at baseline, $kg/m^2/cm$	21.3	24.2	27.0	30.2	73.3	81.8	91.4	100.6
Cases/Participants, N	29/2,185	43/3,190	35/2,642	11/963	19/1,340	44/3,249	42/3,458	13/933
HR (95% CI)	Ref.	0.96 (0.59, 1.56)	0.92 (0.54, 1.54)	0.79 (0.54, 1.54)	Ref.	0.83 (0.47, 1.47)	0.68 (0.36, 1.30)	0.76 (0.35, 1.65)
**45-59 years**								
Mean BMI/WC values at baseline, $kg/m^2/cm$	21.5	24.2	26.9	30.0	76.7	85.4	93.6	102.1
Cases/Participants, N	79/3,825	160/7,350	147/5,744	48/1,880	46/2,636	179/8,333	173/6,544	36/1,286
HR (95% CI)	Ref.	1.02 (0.78, 1.33)	1.18 (0.90, 1.56)	1.19 (0.83, 1.70)	Ref.	1.13 (0.82, 1.57)	1.38 (0.99, 1.92)	1.47 (0.95, 2.29)
**≥60 years**								
Mean BMI/WC values at baseline, $kg/m^2/cm$	21.6	24.5	27.1	30.3	77.9	87.9	96.6	103.2
Cases/Participants, N	92/1,496	176/3,178	151/2,512	49/774	60/1,054	220/3,935	146/2,477	42/494
HR (95% CI)	Ref.	0.93 (0.72, 1.20)	1.03 (0.79, 1.34)	1.15 (0.81, 1.63)	Ref.	0.93 (0.70, 1.24)	0.97 (0.72, 1.32)	1.35 (0.90, 2.01)
**Sex**								
**Female**								
Mean BMI/WC values at baseline, $kg/m^2/cm$	21.2	24.2	27.1	30.4	74.5	83.5	93.1	100.7
Cases/Participants, N	38/2,015	66/3,106	58/2,166	26/836	32/2,145	95/3,907	48/1,795	13/276

HR (95% CI)	Ref.	0.94 (0.63, 1.41)	1.13 (0.74, 1.73)	1.30 (0.78, 2.16)	Ref.	1.25 (0.83, 1.88)	1.20 (0.75, 1.93)	1.98 (1.02, 3.83)*
**Male**								
Mean BMI/WC values at baseline, kg/m^2/cm	21.5	24.3	26.9	30.0	78.1	87.2	95.1	103.0
Cases/Participants, N	161/5,446	308/10,628	278/8,717	85/2,825	106/4,349	359/12,537	286/8,774	81/1,956
HR (95% CI)	Ref.	0.95 (0.79, 1.15)	1.06 (0.87, 1.29)	1.07(0.82, 1.39)	Ref.	1.02 (0.82, 1.26)	1.11 (0.89, 1.39)	1.31 (0.98, 1.75)

Note: BMI, body mass index; WC, waist circumference; HR, Hazard Ratio; CI, confidence interval.

The Cox proportional hazards regression models were used to examine the associations, adjusted for age, sex (not in the sex-stratified analysis), marital status, educational level, monthly income, smoking status, drinking status, physical activity, and sedentary time.

* indicated statistical significance (P<0.05).

Table S3. Sensitivity analyses for the associations of BMI and WC trajectories with the risk of cardiac arrhythmia.

		BMI trajectory		
	**Low-stable**	**Moderate-stable**	**Moderate-high-stable**	**High-stable**
**Model 4**	Ref.	0.99 (0.83, 1.18)	1.12 (0.93, 1.34)	1.17 (0.91, 1.50)
**Model 5**	Ref.	0.96 (0.80, 1.14)	1.08 (0.90, 1.29)	1.13 (0.89, 1.42)
**Model 6**	Ref.	0.96 (0.80, 1.14)	1.08 (0.90, 1.28)	1.12 (0.89, 1.42)
		**WC trajectory**		
	**Low-stable**	**Moderate-stable**	**Moderate-high-stable**	**High-stable**
**Model 4**	Ref.	1.12 (0.88, 1.43)	1.12 (0.87, 1.43)	1.42 (1.06, 1.90)*
**Model 5**	Ref.	1.11 (0.88, 1.41)	1.09 (0.86, 1.39)	1.40 (1.06, 1.86)*
**Model 6**	Ref.	1.11 (0.88, 1.41)	1.09 (0.86, 1.39)	1.40 (1.06, 1.86)*

Note: BMI, body mass index; WC, waist circumference; HR, Hazard Ratio; CI, confidence interval.

The Cox proportional hazards regression models were used to examine the associations.

Model 4 was further adjusted for FBG, HDL, LDL, TC, TG, DBP, and SBP based on Model 3 (N=35,372); Model 5 was further adjusted for family history of CVD based on Model 3 (N=35,739); Model 6 assessed the competing risk from all-cause death, and was adjusted for age, sex, marital status, educational level, monthly income, smoking status, drinking status, physical activity, and sedentary time.

* indicated statistical significance (P<0.05).
